# Advancing Quantitative XRF Analysis of Plant Matrices Through Validation and Extension of Element-Specific Correction Factors by Comparison with INAA Measurements

**DOI:** 10.3390/mps9050142

**Published:** 2026-09-30

**Authors:** Riccardo Fedeli, Konstantin Vergel, Vladimir Galustov, Omari Chaligava, Mirko Legnaro Diamanti, Tiberio Fiaschi, Claudia Angiolini, Mira Aničić Urošević, Inga Zinicovscaia, Stefano Loppi

**Affiliations:** 1 BioAgry Lab, University of Siena, Via Pier Andrea Mattioli 4, 53100 Siena, Italy; m.legnarodiamanti@student.unisi.it (M.L.D.); tiberio.fiaschi@gmail.com (T.F.); angiolini@unisi.it (C.A.); loppi@unisi.it (S.L.); 2 Joint Institute for Nuclear Research, 6 Joliot-Curie Street, 141980 Dubna, Russia; verkn@mail.ru (K.V.); galustov@jinr.ru (V.G.); chaligava@jinr.int (O.C.); inga@jinr.ru (I.Z.); 3 Andronikashvili Institute of Physics, I. Javakhishvili Tbilisi State University, 6 Mikheil Tamarashvili Street, 0186 Tbilisi, Georgia; 4 Institute of Physics, University of Belgrade, Pregrevica 118, 11080 Belgrade, Serbia; mira.anicic@ipb.ac.rs; 5 Horia Hulubei National Institute for R&D in Physics and Nuclear Engineering, 30 Reactorului Street, 077125 Magurele, Romania

**Keywords:** portable X-ray fluorescence, instrumental neutron activation analysis, plant matrices, correction factors, elemental analysis, mosses

## Abstract

X-ray fluorescence (XRF) provides a rapid, non-destructive approach for the multi-element analysis of plant materials, although its quantitative application is limited by matrix-dependent effects that can cause substantial deviations from real elemental concentrations. To compensate for systematic XRF overestimation in plant matrices, element-specific correction factors (CFs) were derived from the ratio between certified elemental concentrations and XRF-measured concentrations in plant certified reference materials (CRMs). Here, the applicability of these CRM-derived CFs to real plant samples was independently validated by comparing corrected XRF results with Instrumental Neutron Activation Analysis (INAA) measurements for 165 moss samples. Application of the CFs markedly improved quantitative agreement, yielding median recoveries close to unity (0.79–1.16) for Zn, K, Ca, Mn, Sr, Rb, Cl, and Fe. The XRF-INAA comparison also enabled the evaluation of additional elements for which CFs had not been established using CRMs. Strong correlations were obtained for Ti and Ni in both XRF analytical modes (ρ = 0.70–0.86), as well as for As in *Soil* mode (ρ = 0.73) and Al in *Geochem* mode (ρ = 0.79), allowing new element-specific CFs to be proposed. Conversely, Ba showed mode-dependent performance, while Cr and V exhibited weak or negligible correlations with INAA, preventing reliable correction. Overall, these findings demonstrate that, for the pXRF instrument and analytical configurations investigated here, CRM-derived CFs can be successfully applied to field-collected moss samples, expand the range of elements that can be reliably quantified under these measurement conditions, and strengthen the potential of pXRF for routine multi-element analysis of plant matrices.

## 1. Introduction

The determination of total elemental composition of plant materials is necessary in several scientific fields, including environmental biomonitoring, agriculture, and food quality assessment [1,2,3]. Accurate multi-element analysis allows the investigation of nutrient dynamics, contamination processes, biogeochemical cycling, and environmental exposure, often requiring the determination of major, minor, and trace elements within the same sample [4]. Consequently, analytical methods capable of rapidly providing reliable multi-element data are increasingly demanded.

Among the available techniques, inductively coupled plasma optical emission spectrometry (ICP-OES) and inductively coupled plasma mass spectrometry (ICP-MS) are generally considered the reference methods for routine multi-element analysis because of their high sensitivity and accuracy [5,6]. However, their routine application is constrained by the need for acid digestion and laboratory facilities, relatively high analytical costs, and the additional time and potential analytical uncertainty associated with sample preparation [7,8]. In this context, portable X-ray fluorescence (pXRF) spectroscopy represents an attractive alternative for rapid multi-element analysis. This technique is non-destructive, requires minimal sample preparation, provides multiple results within seconds, and can be employed both in the laboratory and directly in the field if using a portable instrument [9,10,11].

Owing to these characteristics, XRF has become widely used in geology [12], archaeology [13,14], and in environmental monitoring [15,16], with increasing applications in biological and agricultural studies [17,18,19]. Over the last two decades, pXRF has also been increasingly investigated for the elemental analysis of plant materials, including medicinal plants, crop species, and different plant tissues [20,21,22,23,24,25]. Nevertheless, despite these advantages, quantitative analysis of plant matrices remains particularly challenging since their low density, high organic matter content, and matrix composition strongly influence X-ray absorption and fluorescence emission, frequently leading to systematic deviations from the “*real*” elemental concentrations [26]. Therefore, although pXRF is increasingly applied to plant analysis, obtaining reliable quantitative concentrations remains a major methodological challenge and requires appropriate matrix-specific calibration or correction strategies.

Accordingly, several approaches have been investigated to improve the quantitative performance of pXRF in plant matrices. Early investigations mainly focused on evaluating the feasibility of pXRF through comparison with ICP-based techniques [20,21], whereas later studies investigated matrix-specific calibration procedures [22], empirical regression models [23], and alternative data-processing strategies based on fundamental parameters [27]. Other studies have further evaluated pXRF for nutrient determination in crop species [26,27]. Recently, we proposed element-specific correction factors (CFs) derived from plant certified reference materials (CRMs) to improve the quantitative performance of pXRF for plant matrices [28]. The principal methodological characteristics of these studies are summarized in Table 1.

In our previous study [28], the analytical performance of XRF was evaluated using 12 CRMs, twice the number investigated by Towett et al. [22] (Table 1). Measurements of plant materials by XRF showed a systematic overestimation of elemental concentrations. However, the strong correlations observed between XRF measurements and CRMs enabled the development of element-specific CF, substantially improving the quantitative reliability of XRF measurements for the validated elements (i.e., Ca, Cl, Fe, K, Mn, Rb, Sr, Zn; [28]).

Although the results of Fedeli et al. [28] demonstrated the potential of CF-based quantification, two important limitations remained. Firstly, the CFs were derived exclusively from CRMs, whose elemental composition and concentration ranges may not fully represent those encountered in real plant samples. Consequently, their applicability to routine analyses of field plant matrices has not yet been independently verified. Secondly, several elements detectable by pXRF could not be calibrated because an insufficient number of CRMs provided both suitable reference concentrations and reliable quantitative XRF measurements, as concentrations of these elements were often too low for reliable pXRF quantification. This prevented the establishment and adequate evaluation of robust relationships between pXRF and reference concentrations and, consequently, the development of reliable CFs for these elements.

Instrumental Neutron Activation Analysis (INAA) is particularly suitable for validating portable XRF since both techniques directly analyzed samples without any chemical pre-treatment, in contrast to ICP-based methods, which rely on acid digestion and subsequent liquid solution analysis, thus reducing potential sources of analytical uncertainty associated with digestion efficiency, contamination, or analyte losses [30]. This methodological similarity allows a more direct comparison between the two techniques, while preserving the non-destructive nature of the analysis. However, despite its high analytical reliability, INAA requires access to specialized nuclear facilities and involves irradiation and counting procedures that limit its applicability for rapid, routine analyses. In contrast, pXRF provides multi-element measurements within minutes, requires minimal sample preparation, and can be operated directly in the laboratory or in the field. Therefore, INAA is particularly valuable as an independent reference method for validating XRF, whereas portable XRF represents a more practical tool for routine analysis and large-scale environmental biomonitoring.

Building upon our previous work, we hypothesized that XRF values corrected with our element-specific CFs derived from CRMs, applied to field plant samples are consistent with INAA determinations (used here as an estimate of the “*real*” value) despite the fundamentally different analytical principles of the two techniques, which are however both based on irradiation of untreated samples. We further hypothesized that the direct comparison between pXRF and INAA measurements would allow the derivation of reliable CFs for additional elements that could not be previously calibrated owing the insufficient number of CRMs providing both suitable reference concentrations and reliable pXRF measurements. To test these hypotheses, moss samples collected in the field were analyzed independently by portable pXRF and INAA, providing both external validation of the previously proposed CFs and an extension of the correction framework to additional elements.

## 2. Materials and Methods

### 2.1. Plant Matrices

**Figure 1:**
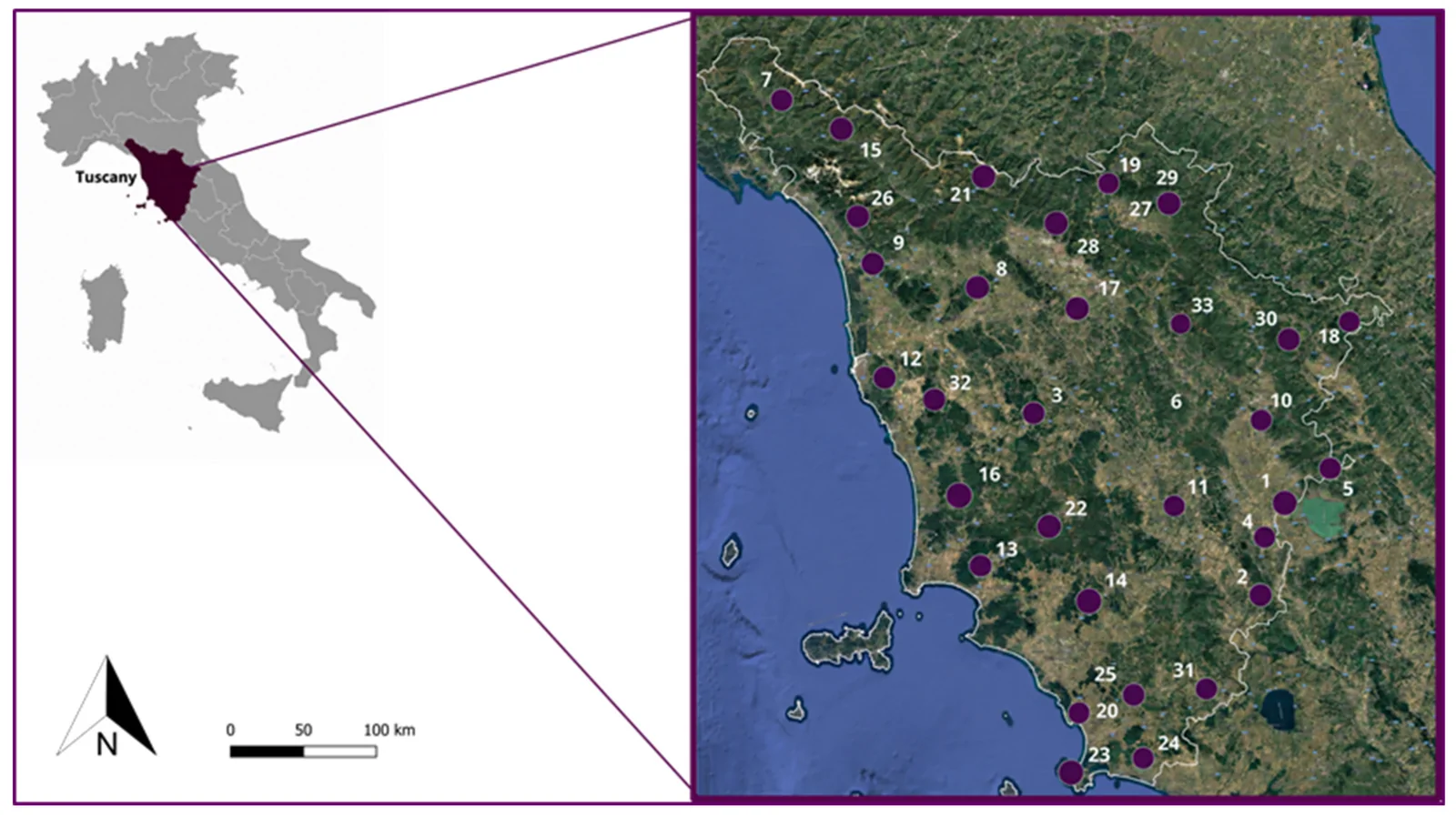
Spatial distribution of sampling sites.

A total of 165 moss samples collected from 33 remote rural sites distributed across Tuscany (Central Italy) were included in the present study. Sampling was carried out between 26 April and 3 May 2025 following the International Cooperative Programme (ICP) Vegetation survey protocol [31]. Sampling sites were located in remote forested areas and were selected to minimize the influence of direct local pollution sources, maintaining appropriate distances from major roads, settlements, industrial and agricultural areas, minor roads, and buildings. At each site, five independent samples of pleurocarp moss (mostly *Hypnum cupressiforme* Hedw., but also *Brachythecium rutabulum* (Hedw.) Schimp., *Eurhynchium striatum* (Hedw.) Schimp., *Pseudoscleropodium purum* Flesich., *Homalothecium lutescens* (Hedw.) H. Rob., *Ctenidium molluscum* (Hedw.) Mitt., *Oxyrrhynchium hians* (Hedw.) Loeske, *Isothecium alopecuroides* (Lam. ex Dubois) Isov.) were randomly collected within a 50 × 50 m sampling area, resulting in a total of 165 samples (Figure 1).

**Figure 2:**
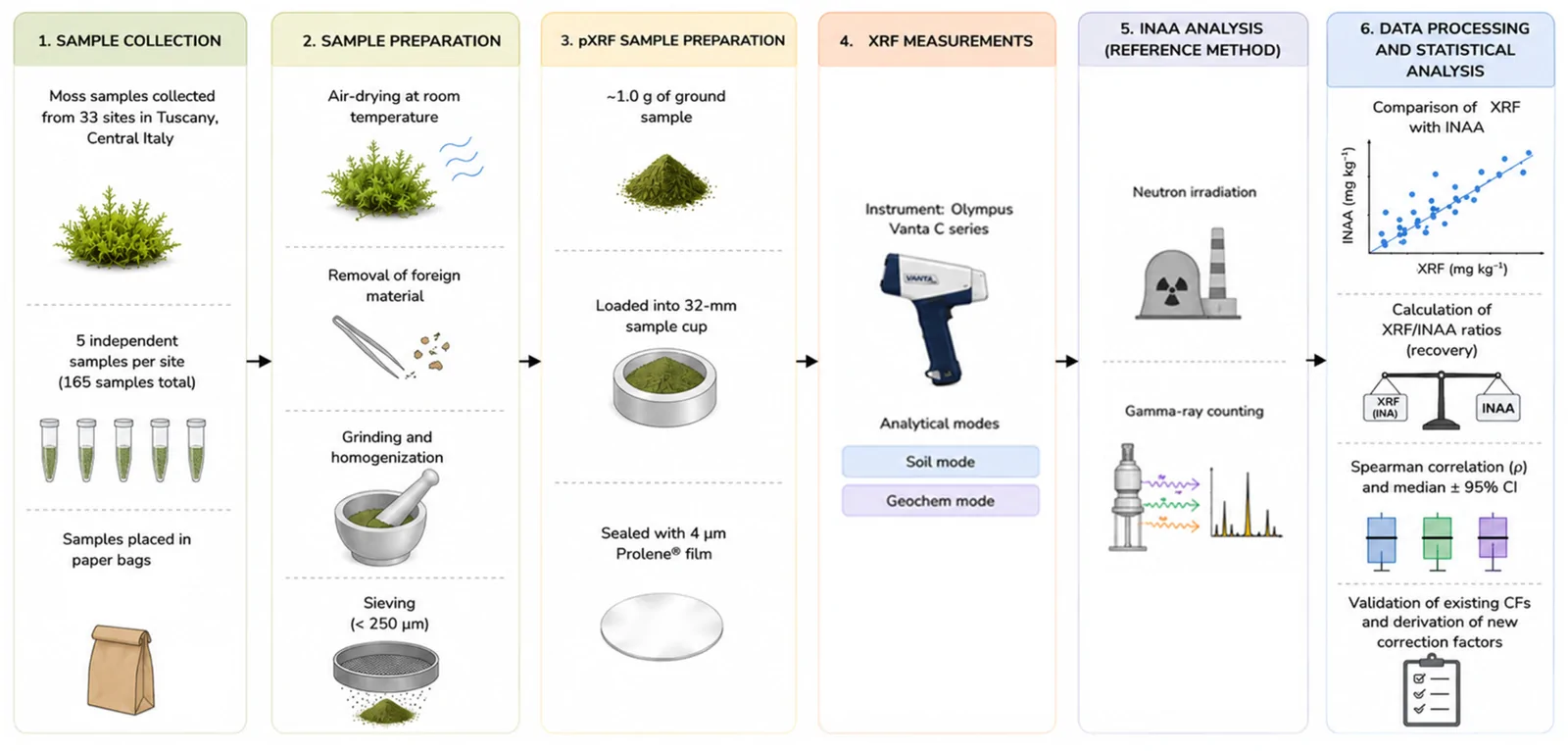
Experimental flow of the study.

Samples were put in paper bags, air-dried and stored for subsequent preparation and analysis. The resulting dataset was used for the comparative evaluation of XRF and INAA measurements and for the independent validation and extension of the element-specific CFs (Figure 2).

### 2.2. XRF Analysis

The concentrations of major, minor and trace elements were determined following the analytical procedure described by Fedeli et al. [28]. Approximately 1.0 g of ground (<2 mm) moss material was transferred into a 32 mm XRF sample cup and sealed with a 4 μm Prolene^®^ film (Ethicon, Inc., Raritan, NJ, USA). Samples were analysed using a portable X-ray fluorescence spectrometer (Vanta C series, Olympus, Waltham, MA, USA) operating in both *Soil* and *Geochem* analytical modes. *Soil* and *Geochem* are manufacturer-specific factory analytical configurations and do not represent standardized XRF measurement protocols. The two modes differ in their excitation conditions, quantification algorithms, and reportable elemental suites. They were both investigated because their different analytical configurations provide complementary elemental coverage and allowed us to evaluate the performance of the instrument under the factory modes available for plant analysis.

The *Soil* mode uses a factory empirical calibration based on Compton normalization, whereas the *Geochem* mode uses a Fundamental Parameters (FP) algorithm that accounts for inter-element matrix effects. Thus, the concentrations reported by the two modes are generated using different quantification approaches.

Each analytical mode employs three sequential excitation beams with an acquisition time of 20 s per beam. The excitation conditions and spectral lines relevant to the interpretation of Ba, V, and Cr differed between beams. In *Soil* mode, Ba is primarily quantified from its Kα lines in Beam 1 at 50 keV using a 350 μm Cu filter. V and Cr are quantified in Beam 2 at 40 keV using a 2000 μm Al filter. Ba is also measured in the 40 keV beam so that Ba-related spectral interference can be accounted for in the calculation of V and Cr concentrations. In *Geochem* mode, Ba Lβ lines are measured in the 40 keV beam using the 2000 μm Al filter, whereas Ba Kα lines are measured in the 50 keV beam using the 350 μm Cu filter. The latter provides the primary signal for Ba quantification, with matrix, absorption, and cross-beam corrections applied by the instrumental algorithm.

The elemental suites reported by the instrument also differ between the two factory modes. Consequently, not all elements were available for quantitative evaluation in both modes; for example, Al was reported only in *Geochem* mode, whereas As, Cr, and V were reported only in *Soil* mode in the present dataset. Therefore, the absence of an element from one mode does not necessarily indicate unsuccessful quantification but may reflect the element-specific output of the corresponding factory analytical configuration. Elemental concentrations are reported as mg kg^−1^ dry weight (DW).

### 2.3. INAA

The elemental composition of ground (<2 mm) moss samples was determined via instrumental neutron activation analysis at the Regata facility of the IBR-2 reactor (Joint Institute for Nuclear Research, Dubna). To determine elements producing short-lived radionuclides (Al, Mg, Cl, Ca, Ti, V, and Mn), samples were irradiated for 3 min at a thermal neutron flux of 1.2 × 10^12^ n cm^−2^ s^−1^ and counted immediately after irradiation for 15 min. To determine mass fractions of Na, K, Sc, Cr, Fe, Co, Ni, Zn, As, Br, Rb, Sr, Sb, Cs, Ba, La, Ce, Sm, Tb, Yb, Hf, Ta, W, Th, and U, samples were irradiated for 3 days at a neutron flux of 1.1 × 10^11^ n cm^−2^ s^−1^. Following irradiation, the samples were repacked and measured twice: after 4 days and after 20 days, with counting times of 30 min and 1.5 h, respectively. Gamma spectra of induced activities were acquired using Canberra HPGe detectors with efficiencies of 70–100% and resolutions of 1.8–2.0 keV at the ^1332^ keV ^60^Co full-energy peak. Spectral analysis was performed with Genie2000 software (version 4.0, Canberra), and elemental mass fractions were calculated using software developed at FLNP [32].

A set of reference materials, including NIST 2709a—San Joaquin Soil, NIST 2709—San Joaquin Soil, NIST 1515—apple leaves, NIST 1547—peach leaves, NIST 2710—Montana Soil, NIST 1633b—bituminous coal fly ash, NIST 2711a—Montana II Soil, BCR-667 Estuarine Sediment, and CTA-FFA-1—fine fly ash, were irradiated with samples to create a group standard and to ensure the quality of the analysis [33,34,35,36,37,38,39,40,41]. According to Greenberg et al. [42], chemical matrix effects, known to be significant sources of error in some other types of instrumental chemical analysis, are insignificant in INAA. The quality control results of NAA along with limits of detection (LOD) are given in Table S1.

### 2.4. Recoveries and Statistical Analysis

For the validation of previously developed CFs, recovery was calculated as the ratio between the concentration determined by XRF, corrected using the CFs suggested by Fedeli et al. [28] and the corresponding concentration measured by INAA for each individual sample. Recovery values, intended as ratios of XRF to INAA (used here as an estimate of the “*real*” value), are reported as median ± uncertainty (%), where uncertainty is defined as the 95% confidence interval (CI) of the median, calculated through 1000-iteration bootstrapping and divided by the median. The number of paired observations varied among elements and analytical modes because, in some samples, the concentration of a given element was below the limit of detection (LOD) of either XRF or INAA. Statistical analyses were therefore performed independently for each element and analytical mode, using only samples for which valid paired XRF-INAA measurements were available. For elements that could not be calibrated in the previous study (i.e., Al, As, Ba, Cr, Ni, Ti, and V), new CFs have been calculated as the median of the individual XRF/INAA ratios obtained from all paired observations available for each element. The element-specific CF was calculated as:

*CF* = *median*(*C*_*XRF*/*C*_*reference*),

where *C_XRF* is the concentration measured by *pXRF* and *C_reference* is the corresponding reference concentration (certified CRM concentration for the previously developed CFs or INAA concentration for CFs derived in the present study). For routine application, the corrected XRF concentration is obtained as:

*C*_*corrected* = *C*_*XRF*/*CF*

Thus, the appropriate element- and analytical-mode-specific CF is selected, and the raw XRF concentration is divided by this factor. A complete list of the previously developed (Fedeli et al. [28] and newly derived CFs, together with their applicable analytical modes, is provided in Table S2.

The relationship between XRF and INAA measurements was assessed using Spearman’s rank correlation coefficient (ρ). In addition, ordinary least-squares linear regression was performed on the uncorrected XRF concentrations, using XRF concentration as the dependent variable and INAA concentration as the independent variable. The slope and intercept of the fitted regression lines were used to assess proportional and constant bias, respectively. Descriptive statistics were calculated separately for the original and CF-corrected XRF concentrations for each element and analytical mode, including sample size (n), mean, median, minimum and maximum values, first and third quartiles (Q1 and Q3), 5th and 95th percentiles (P5 and P95), range, interquartile range (IQR), standard deviation (SD), mean absolute deviation (MAD), and coefficient of variation (CV). The complete descriptive statistical summary is provided in Table S3. All statistical analyses were performed using R software (version 4.5.2; [43]).

## 3. Results and Discussion

### 3.1. Independent Validation of Previously Developed CFs

In our previous study [28], CFs were derived from CRMs to offset the systematic overestimation observed for pXRF measurements. In the present study, their applicability was evaluated using 165 moss samples analysed by INAA, providing an external validation under realistic analytical conditions. Moreover, to the best of our knowledge, this is the first study attempting to calibrate pXRF for the quantitative elemental analysis of moss samples.

Using our CFs, XRF measurements (Table 2) showed recovery values ranging 79–116% for both *Soil* and *Geochem* modes (i.e., Zn (0.79–0.81), K (0.88), Ca (1.02–1.06), Mn (0.95–1.14), Sr (0.90–1.05), Rb (0.88–0.97), Cl (1.13–1.16) and Fe (1.01–1.11), with very small uncertainties, supporting the applicability of the CRM-derived CFs to field-collected moss samples analysed using the same pXRF system and analytical configurations.

Although the number of valid paired observations varied among elements and analytical modes (*n* = 130–163), the sample size remained relatively high in all cases. Since each element and analytical mode was evaluated independently, differences in *n* did not affect comparisons across elements, as recoveries and correlations were calculated separately using the available paired observations. Moreover, the relatively large sample sizes, together with the small uncertainties associated with the median recovery values, support the robustness of the validation of the element-specific CFs. These findings extend the conclusions of Towett et al. [22], who demonstrated that quantitative XRF analysis of plant materials requires matrix-specific calibration by showing that such calibration can be successfully transferred from CRMs to real field samples. Similarly, previous studies [20,21,23,25] comparing XRF data with ICP-based techniques reported a general good agreement, as they relied on regression models. The present work demonstrates that a simple CF-based approach can achieve comparable quantitative performance while maintaining excellent agreement with an independent reference technique.

### 3.2. Development of CFs for Additional Elements

A major limitation of our previous study [28] was the impossibility of developing CFs for several elements since certified concentrations were not present in the available CRMs. Based on the very good agreement between pXRF and INAA results for the elements with available CFs, the comparison with INAA for other elements allowed us to overcome this limitation. Indeed, Ti and Ni exhibited strong correlations between the two techniques both in *Soil* (ρ = 0.86 and ρ = 0.78, respectively) and *Geochem* (ρ = 0.79 and 0.70, respectively) modes, as well as very small uncertainties of the recovery values. Aluminium in *Geochem* mode and As in *Soil* mode also showed strong correlations with INAA (ρ = 0.79 and 0.73, respectively) and very small uncertainties of the recovery values. The slope and intercept of the fitted regression lines provided additional information on the nature of the discrepancies between pXRF and INAA measurements (Table 3). For Ti and Ni, slopes greater than unity were observed in both analytical modes (2.553 and 2.905 in *Soil* mode and 5.289 and 3.017 in *Geochem* mode, respectively), indicating a marked proportional component of the XRF bias. For Ni, the intercepts were close to zero (−3.60 and −9.63 mg kg^−1^ in *Soil* and *Geochem* modes, respectively), suggesting that the discrepancy between XRF and INAA was predominantly proportional over the investigated concentration range. As in *Soil* mode similarly showed a slope greater than unity (2.129) with a relatively small intercept (6.63 mg kg^−1^). In contrast, Ti showed positive intercepts (124.67 and 534.63 mg kg^−1^ in *Soil* and *Geochem* modes, respectively), while Al in *Geochem* mode exhibited a particularly large positive intercept (10,794.03 mg kg^−1^), indicating that a constant component may also contribute to the observed bias. Overall, these regression parameters complement the median XRF/INAA ratios by distinguishing proportional from constant components of the analytical discrepancy and highlight cases in which a single multiplicative CF may not fully account for the observed bias.

**Figure 3:**
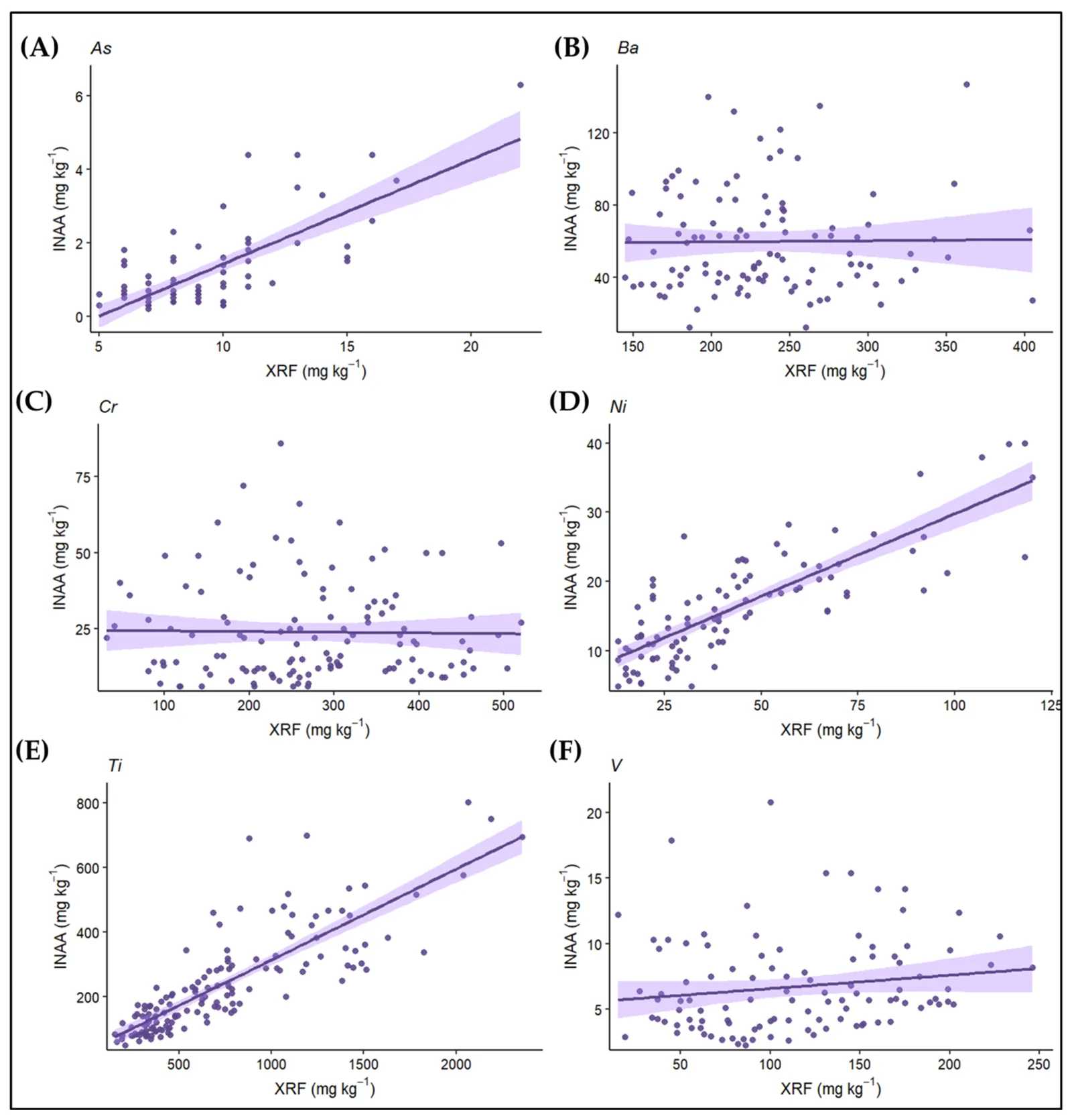
Relationship between elemental concentrations determined by X-ray fluorescence (XRF) and Instrumental Neutron Activation Analysis (INAA) for the additional elements investigated in *Soil* mode: (**A**) As, (**B**) Ba, (**C**) Cr, (**D**) Ni, (**E**) Ti, and (**F**) V. Each point represents an individual sample for which paired XRF and INAA measurement was available. Solid lines represent the fitted linear relationships between XRF and INAA concentrations, while shaded areas indicate the corresponding 95% confidence intervals.

**Figure 4:**
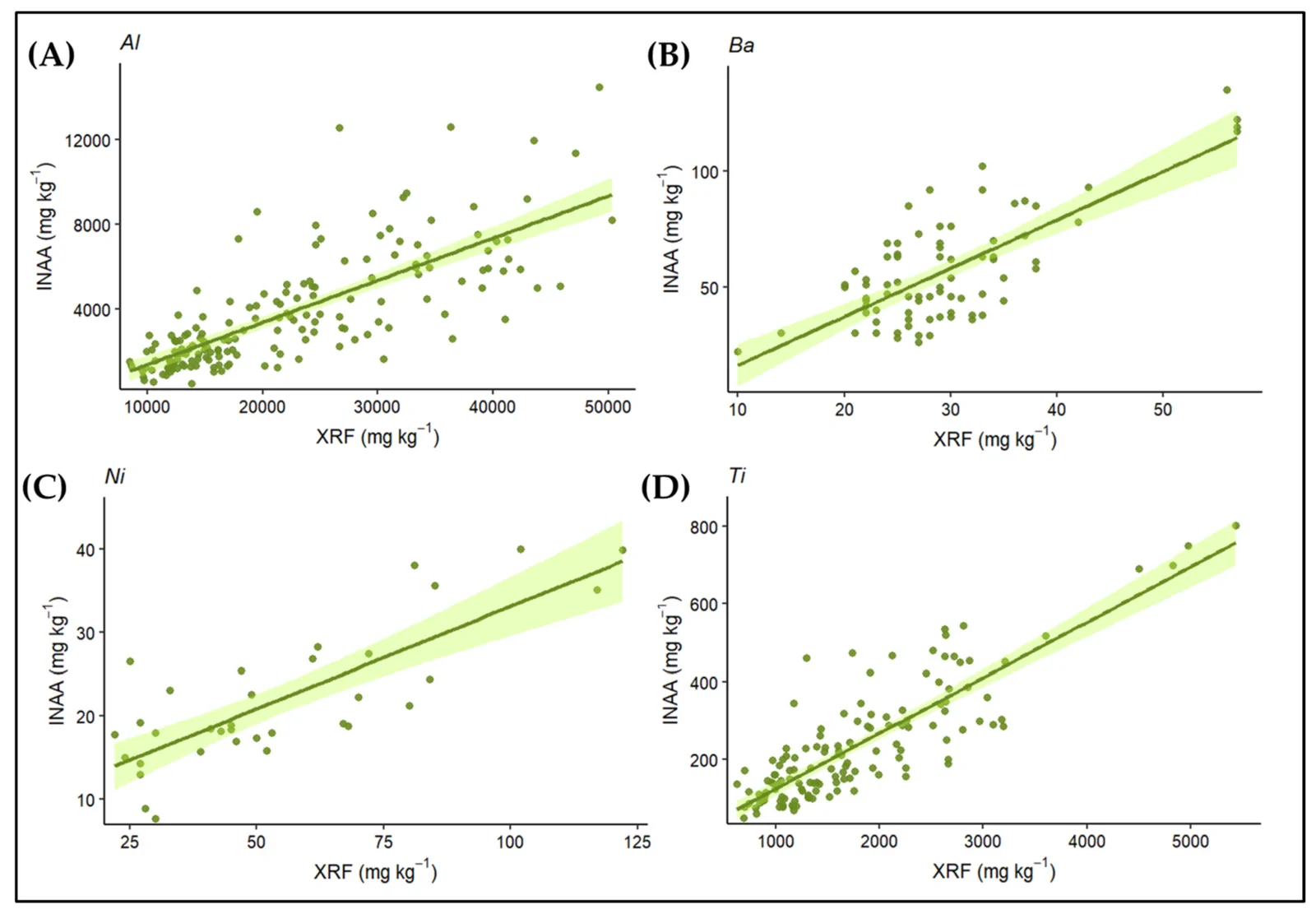
Relationship between elemental concentrations determined by X-ray fluorescence (XRF) and Instrumental Neutron Activation Analysis (INAA) for the additional elements investigated in *Geochem* mode: (**A**) Al, (**B**) Ba, (**C**) Ni, and (**D**) Ti. Each point represents an individual sample for which paired XRF and INAA measurement was available. Solid lines represent the fitted linear relationships between XRF and INAA concentrations, while shaded areas indicate the corresponding 95% confidence intervals.

Barium showed a different behaviour depending on the analytical mode: while a moderate correlation (ρ = 0.52) with a very strong statistical significance was found in *Geochem* mode, no correlation with INAA emerged in *Soil* mode (ρ = −0.10). These results indicate that the analytical bias observed for these elements is predominantly or largely systematic, and it can therefore be effectively compensated using element-specific CFs. Vanadium showed a very weak correlation (ρ = 0.21), and Cr showed no statistically significant correlation at all, indicating that their analytical variability cannot be explained solely by a systematic bias (Table 3; Figure 3 and Figure 4). Accordingly, the slope and intercept values obtained for Ba, Cr, and V should be interpreted with caution because of their weak or mode-dependent relationships with INAA and do not provide additional support for the application of reliable CFs to these elements. The weak relationships observed for V and Cr may also reflect the complexity of spectral deconvolution under the *Soil* analytical configuration. As described in Section 2.2, Ba is measured in the same 40 keV beam used for V and Cr so that Ba-related spectral interference can be accounted for by the instrumental algorithm. Nevertheless, residual spectral and matrix-dependent effects cannot be excluded in a low-density plant matrix and may have contributed to the poor agreement with INAA, further limiting the applicability of a simple multiplicative CF to V and Cr.

### 3.3. Comparison with Previous Studies

Previous studies validating pXRF for plant analysis have mostly compared XRF measurements with ICP-OES or ICP-MS analysis, after acid digestion of the samples [21,22,24,26]. Although ICP-based techniques provide excellent analytical sensitivity, they require extensive chemical pretreatment, including acid digestion, dilution and solution handling. These additional steps inevitably introduce potential sources of analytical uncertainty associated with incomplete mineralisation, contamination and analyte loss.

In contrast, both pXRF and INAA rely on the direct analysis of samples, without any chemical pre-treatment. Consequently, both techniques evaluate exactly the same physical material, minimising uncertainties associated with sample preparation. This methodological similarity makes INAA a particularly suitable independent reference technique for validating quantitative XRF measurements.

Towett et al. [22] emphasised the importance of matrix-specific calibration in XRF analysis, and Zhou et al. [44] identified the absence of standardised calibration strategies as one of the principal limitations preventing wider quantitative application of XRF in plant science. The present work complements these studies by demonstrating that CFs developed from XRF analysis of CRMs can be independently validated using a completely different analytical principle. Unlike previous regression-based calibration approaches, the CF strategy adopted here is straightforward to implement and requires only a division by an element-specific coefficient. This simplicity facilitates routine application without requiring recalibration models or complex statistical procedures, while maintaining satisfactory agreement with the reference method.

### 3.4. Implications for Quantitative XRF Analysis of Plant Matrices

The combination of the present work with our previous study [28] establishes a methodological framework for deriving and validating instrument- and mode-specific CFs for quantitative XRF analysis of plant matrices. Initially, CFs were derived from CRMs, subsequently, these CFs were independently validated on a large dataset of real field moss samples using INAA. Lastly, new CFs were provided for additional elements by comparison with INAA measurements. However, it should be noted that in this study, moss samples were investigated, which are indeed part of the plant kingdom, but are very simple organisms, lacking real tissues and a vascular system and adsorb water and mineral elements throughout their whole surface. An additional methodological consideration concerns sample mass and thickness, particularly for *Geochem* measurements based on the FP algorithm. Approximately 1.0 g of ground moss was analysed in a 32 mm sample cup in the present study. Because plant materials have relatively low bulk density, variations in sample thickness and packing density can affect X-ray attenuation and, consequently, quantitative XRF results. Although the same sample preparation procedure was consistently applied to all samples, conditions of effective infinite thickness were not experimentally verified. Sample mass, thickness, and packing density should therefore be considered additional sources of uncertainty and should be specifically evaluated in future applications of FP-based quantification to plant matrices. Nevertheless, overall, our results demonstrate that XRF can provide reliable quantitative information for a substantial number of macro- and microelements when appropriate element-specific CFs are applied. In this context, INAA should be regarded as a robust independent reference technique for validation, whereas the rapidity, portability, minimal sample preparation, and straightforward application of the CF approach make pXRF more suitable for routine and large-scale analysis of plant materials. Although some elements still require further methodological improvement, the present study demonstrates the potential of an element-specific CF approach to improve quantitative XRF analysis of plant matrices. The CF values reported here are specific to the XRF instrument and analytical modes used in this study and should therefore not be considered universally applicable to other XRF systems. Rather, the methodological framework proposed here can be adapted to other instruments by deriving and validating system- and mode-specific CFs using appropriate reference materials and independent reference measurements. This approach may facilitate the broader implementation of quantitative XRF analysis in environmental biomonitoring, agriculture, plant nutrition, and ecological research.

## 4. Conclusions

This study provides an independent validation of previously developed element-specific correction factors (CFs) for the quantitative XRF analysis of plant matrices using INAA measurements of field-collected moss samples. The application of CRM-derived CFs substantially improved the agreement between XRF and INAA measurements for Zn, K, Ca, Mn, Sr, Rb, Cl, and Fe, supporting their transferability from certified reference materials to real plant samples. In addition, the comparison with INAA allowed the correction approach to be extended to additional elements, with promising results for Ti and Ni in both analytical modes, Al in *Geochem* mode, and As in *Soil* mode. Conversely, the poor relationships observed for V and Cr indicate that a simple element-specific correction factor is not sufficient for reliable quantification of these elements, while Ba showed a marked mode-dependent performance.

Overall, the independent validation of CRM-derived CFs on field samples and the extension of the approach to additional elements strengthen the potential of pXRF for a rapid, non-destructive, routine multi-element analysis of plant materials. Further validation across a wider range of plant matrices and analytical conditions will be important to broaden the applicability of this approach and support the development of standardized procedures for quantitative pXRF analysis of plant samples.

## Figures and Tables

**Table 1:** Summary of previous studies evaluating X-ray fluorescence (pXRF) for quantitative elemental analysis of plant matrices, including sample type and number, investigated matrix, reference analytical method, number of certified reference materials (CRMs), and correction factors (CFs).

*Type*	*n*	*Matrix*	*Reference Method*	*CRM (n)*	*CFs*	*Reference*
Field Samples	5	Medicinal plants	ICP-AES/ICP-MS	x	x	Queralt et al. [20]
Field Samples	4	Crop plants	ICP-OES	x	x	McLaren et al. [21]
CRMs	6	Plant CRMs *	CRMs	6	x	Towett et al. [22]
Field Samples	28	Crop plants	ICP-OES	3	x	Borges et al. [23]
Field Samples	n.r.	Crop plants	-	x	x	Montanha et al. [29]
CRMs	12	Plant CRMs ^#^	CRMs	12	12	Fedeli et al. [28]
Field Samples	24	Tropical plants	ICP-OES	x	x	El Mellouki et al. [25]
Field Samples	144	Plant tissues	ICP-AES	x	x	Zhang et al. [26]
Field Samples	165	Moss	INAA	x	17	This study

* = NIST 1515, 1547, 1568a, 1570a, 1573a, 1575a; ^#^ = BCR-1135, GBW07603, GBW07604, IAEA-336, IAEA-392, M1, M2, M3, NFA-36, NIST SRM 1515, NIST SRM 1547, NIST SRM 1573a; n.r. = not reported.

**Table 2:** Comparison between corrected portable X-ray fluorescence measurements and Instrumental Neutron Activation Analysis (INAA) for the elements previously calibrated by Fedeli et al. [28], using *Soil* and *Geochem* modes.

Element	*Soil* Mode			*Geochem* Mode		
	*n*	ρ	Median ± U	*n*	ρ	Median ± U
Ca	158	0.81 ***	1.06 ± 3.2	158	0.69 ***	1.02 ± 6.4
Cl	160	0.67 ***	1.13 ± 8.0	161	0.42 ***	1.16 ± 11.6
Fe	153	0.91 ***	1.11 ± 5.0	153	0.92 ***	1.01 ± 6.2
K	160	0.76 ***	0.88 ± 6.5	-	-	-
Mn	163	0.75 ***	0.95 ± 10.3	161	0.90 ***	1.14 ± 3.3
Rb	152	0.87 ***	0.88 ± 4.1	130	0.83 ***	0.97 ± 5.1
Sr	147	0.74 ***	1.05 ± 7.0	145	0.78 ***	0.90 ± 5.6
Zn	163	0.89 ***	0.81 ± 3.5	153	0.87 ***	0.79 ± 4.6

*n* indicates the number of paired XRF-INAA measurements available for each element, and ρ indicates the Spearman’s rank correlation coefficient between XRF and INAA concentrations. U indicates uncertainty (expressed as %). Corrected values were obtained by applying the element-specific correction factors developed by Fedeli et al. [28]—indicates that the element was not quantified in the corresponding analytical mode. *** = *p*-value < 0.001.

**Table 3:** Comparison between portable X-ray fluorescence (XRF) and Instrumental Neutron Activation Analysis (INAA) for the additional elements investigated in *Soil* and *Geochem* analytical modes.

Element	*Soil* Mode					*Geochem* Mode				
	*n*	ρ	Median ± U	Slope	Intercept	*n*	ρ	Median ± U	Slope	Intercept
Al	-	-	-	-	-	165	0.79 ***	6.52 ± 1.1	3.031	10,794.03
As	78	0.73 ***	10.80 ± 1.4	2.129	6.63	-	-	-	-	-
Ba	105	−0.10	4.38 ± 4.5	0.485	206.81	77	0.52 ***	0.52 ± 29.0	0.287	12.97
Cr	124	0.003	14.14 ± 1.0	1.322	243.1	-	-	-	-	-
Ni	97	0.78 ***	2.59 ± 3.1	2.905	−3.6	34	0.70 ***	2.43 ± 5.9	3.017	−9.63
Ti	144	0.86 ***	3.16 ± 2.1	2.553	124.67	137	0.79 ***	7.79 ± 1.0	5.289	534.63
V	106	0.21 *	18.41 ±1.0	5.739	76.97	-	-	-	-	-

*n* indicates the number of paired XRF-INAA measurements available for each element, and ρ indicates the Spearman’s rank correlation coefficient between the two analytical techniques. For each sample, the XRF/INAA concentration ratio was calculated, and the median of the individual ratios was used to derive the element-specific correction factor (CF). U indicates uncertainty (expressed as %)—indicates that the element was not quantified in the corresponding analytical mode. Slope and intercept (mg kg^−1^) refer to ordinary least-squares linear regressions performed on the uncorrected data, with XRF concentration as the dependent variable and INAA concentration as the independent variable. Slope and intercept were used to characterize proportional and constant bias, respectively. * = *p*-value <0.05; *** = *p*-value < 0.001.

## Data Availability

Data are available on reasonable request from the corresponding author.

## Supplementary Materials

The following supporting information can be downloaded at https://www.mdpi.com/article/10.3390/mps9050142/s1, Table S1. Quality assurance of neutron activation analysis at the Regata facility, in mg kg^−1^; Table S2. Element-specific correction factors (CFs) for XRF quantification of plant matrices in *Soil* and *Geochem* analytical modes. Previously developed CFs were derived from plant certified reference materials (CRMs) as the median ratio between XRF-measured and certified concentrations [28], whereas newly proposed CFs were derived in the present study as the median ratio between uncorrected XRF and INAA concentrations measured in field-collected moss samples; Table S3. Descriptive statistics of original (uncorrected) and CF-corrected XRF concentrations for each element in *Soil* and *Geochem* analytical modes. Statistics include sample size (n), mean, median, minimum, maximum, first and third quartiles (Q1 and Q3), 5th and 95th percentiles (P5 and P95), range, interquartile range (IQR), standard deviation (SD), mean absolute deviation (MAD), and coefficient of variation (CV). Concentrations are expressed as mg kg^−1^ dry weight.
