# Supplementary material for: Advancing Quantitative XRF Analysis of Plant Matrices Through Validation and Extension of Element-Specific Correction Factors by Comparison with INAA Measurements
Source: Methods Protoc. 2026 Sep 30;9(5):142. doi: 10.3390/mps9050142 (PMC13707647; doi:10.3390/mps9050142)
Supplement: Supplementary file 1 [file mps-09-00142-s001.zip › mps-4555054-supplementary.pdf]

Table S1. Quality assurance of neutron activation analysis at the Regata facility, in mg·kg^{-1}

	SRM	Determined	Uncertainty %	Certified	Uncertainty %	LOD
Na	2709	9684	4.1	11600	2.6	20
Mg	1515	2699	4.4	2710	3	100
Al	2710	65319	5.3	64400	1.2	40
Cl	1547	347	8.2	360	5.3	44
K	1515	15674	8	16100	1.2	980
Ca	1515	14518	14.5	15260	1	70
Sc	2709	11.9	5.5	12	3	0.005
Ti	2709a	3116	6.8	3360	2.1	30
V	1632c	23.9	4.6	23.72	2.2	0.11
Cr	667	189	6.3	178	9	1.3
Mn	1633b	132.5	8.3	131.8	1.3	0.38
Fe	667	46198	5.4	44800	2.2	21
Co	667	23.7	5.5	23	5.6	0.01
Ni	667	134.9	6.8	128	7	0.48
Zn	667	177.6	4.4	175	7.4	0.4
As	2711a	102	6	107	5	0.14
Br	667	82.8	4.4	99.7	2.5	0.1
Rb	2711a	126	16.6	120	3	0.09
Sr	2709	244	9.2	231	0.9	1.4
Sb	667	1.031	5.1	0.96	5.2	0.002
Cs	667	8.6	3	7.8	9	0.005
Ba	FFA-1	792	11.1	835	6.7	1.2
La	FFA-1	61.3	3.8	60.7	6.6	0.03
Ce	FFA-1	122	5.6	120	5.8	0.04
Sm	FFA-1	8.2	17	10.9	5.5	0.001
Tb	FFA-1	1.38	10.1	1.39	10.5	0.002
Hf	FFA-1	7.3	6.1	6.2	7.4	0.01
Yb	FFA-1	4.24	4.4	4.16	5.1	0.002
Ta	FFA-1	8.3	6.1	6.2	7.4	0.002
W	FFA-1	10.5	11.1	10.2	10.5	0.001
Th	667	10.2	4.7	10	5	0.005
U	2711a	3.007	8.5	3.01	4	0.003

Table S2. Element-specific correction factors (CFs) for XRF quantification of plant matrices in Soil and Geochem analytical modes. Previously developed CFs were derived from plant certified reference materials (CRMs) as the median ratio between XRF-measured and certified concentrations [28], whereas newly proposed CFs were derived in the present study as the median ratio between uncorrected XRF and INAA concentrations measured in field-collected moss samples.

Element	Soil mode CF	Geochem mode CF	Derivation	Source
Ca	2.52	4.87	Plant CRMs (XRF/certified value)	Fedeli et al. [28]
Cl	3.28	7.80	Plant CRMs (XRF/certified value)	Fedeli et al. [28]
Fe	3.63	4.29	Plant CRMs (XRF/certified value)	Fedeli et al. [28]
K	3.26	–	Plant CRMs (XRF/certified value)	Fedeli et al. [28]
Mn	5.86	4.89	Plant CRMs (XRF/certified value)	Fedeli et al. [28]
Rb	1.44	1.15	Plant CRMs (XRF/certified value)	Fedeli et al. [28]
Sr	2.95	1.23	Plant CRMs (XRF/certified value)	Fedeli et al. [28]
Zn	3.43	3.88	Plant CRMs (XRF/certified value)	Fedeli et al. [28]
Al	–	6.52	Moss samples (XRF/INAA)	Present study
As	10.80	–	Moss samples (XRF/INAA)	Present study
Ni	2.59	2.43	Moss samples (XRF/INAA)	Present study
Ti	3.16	7.79	Moss samples (XRF/INAA)	Present study

CFs are applied according to $C\_corrected = C\_XRF / CF$. "–" indicates that no CF was established for the corresponding analytical mode. The reported CFs are specific to the XRF instrument and analytical modes used in these studies and should not be considered universally applicable to other XRF systems.

Table S3. Descriptive statistics of original (uncorrected) and CF-corrected XRF concentrations for each element in Soil and Geochem analytical modes. Statistics include sample size (n), mean, median, minimum, maximum, first and third quartiles (Q1 and Q3), 5th and 95th percentiles (P5 and P95), range, interquartile range (IQR), standard deviation (SD), mean absolute deviation (MAD), and coefficient of variation (CV). Concentrations are expressed as mg kg^{-1} dry weight.

Element	XRF mode	Dataset	n	Mean	Median	Min	Max	Q1	Q3	P5	P95	Range	IQR	SD	MAD	CV(%)
Fe	Soil	Original	153	10738	8840	1523	32034	5525	14603	3645	24193	30511	9078	6775	5401	63
Fe	Soil	Corrected	153	2954	2435	420	8825	1496	4023	1004	6665	8405	2527	1870	1491	63
Zn	Soil	Original	163	89	82	39	219	66	109	51	146	180	43	32	25	36
Zn	Soil	Corrected	163	26	24	11	64	19	32	15	43	52	13	9	7	36
K	Soil	Original	160	18646	17780	8115	36562	14709	21865	11295	29405	28447	7156	5586	4386	30
K	Soil	Corrected	160	5671	5389	2783	11215	4441	6477	3473	9078	8433	2036	1684	1296	30
Ca	Soil	Original	158	26034	23181	8265	67731	18600	31084	13503	47572	59466	12484	10829	8279	42
Ca	Soil	Corrected	158	10397	9216	3280	26877	7382	12434	5361	18962	23598	5052	4363	3340	42
Mn	Soil	Original	163	864	777	275	2237	598	1030	419	1695	1962	432	381	291	44
Mn	Soil	Corrected	163	146	132	47	382	102	176	72	287	335	74	64	49	44
Cl	Soil	Original	160	1064	969	334	2510	745	1253	530	1905	2176	508	434	336	41
Cl	Soil	Corrected	160	324	295	102	765	227	382	161	581	663	155	132	102	41
Sr	Soil	Original	147	112	106	42	218	87	134	63	174	176	47	35	28	31
Sr	Soil	Corrected	147	38	36	14	74	29	46	21	59	60	16	12	9	31
Rb	Soil	Original	152	15	14	4	45	9	19	6	28	41	10	8	6	51
Rb	Soil	Corrected	152	10	9	3	31	6	13	4	20	28	7	5	4	51
Fe	Geochem	Original	153	11277	9469	1847	30006	6178	15184	4213	23968	28159	9006	6463	5270	57
Fe	Geochem	Corrected	153	2623	2217	431	6994	1448	3458	987	5575	6564	2010	1494	1210	57
Zn	Geochem	Original	153	97	89	47	230	71	115	52	172	183	44	35	28	36
Zn	Geochem	Corrected	153	25	23	12	59	18	30	13	44	47	11	9	7	36
Ca	Geochem	Original	158	46672	43721	21921	101452	35941	54526	27754	75274	79531	18586	15169	11686	33
Ca	Geochem	Corrected	158	9584	8978	4501	20832	7380	11196	5699	15457	16331	3816	3115	2400	33
Mn	Geochem	Original	161	878	715	202	2875	527	1123	304	2085	2673	596	540	412	61
Mn	Geochem	Corrected	161	180	146	41	588	108	230	62	426	547	122	110	84	61
Cl	Geochem	Original	161	2542	2313	296	8013	1515	3280	630	5364	7717	1765	1477	1128	58
Cl	Geochem	Corrected	161	326	297	38	1027	194	421	81	688	989	226	189	145	58
Sr	Geochem	Original	145	39	37	16	79	31	45	22	68	63	14	13	10	33

Sr	Geochem	Corrected	145	32	30	13	64	25	37	18	55	51	11	11	8	33
Rb	Geochem	Original	130	14	12	5	46	8	18	6	27	41	10	8	6	54
Rb	Geochem	Corrected	130	12	10	4	40	7	16	5	23	36	9	7	5	54
